# Trajectories of disability throughout early life and labor force status as a young adult: Results from the Longitudinal Study of Australian Children

**DOI:** 10.5271/sjweh.3994

**Published:** 2022-02-25

**Authors:** Marissa Shields, Matthew J Spittal, Stefanie Dimov, Anne Kavanagh, Tania L King

**Affiliations:** 1 Disability and Health Unit, Centre for Health Equity, Melbourne School of Population and Global Health, The University of Melbourne, Victoria, Australia; 2 Centre for Mental Health, Melbourne School of Population and Global Health, The University of Melbourne, Victoria, Australia

**Keywords:** employment, group-based trajectory modeling, young people

## Abstract

**Objectives:**

Young people with disabilities have poorer labor force outcomes than their peers without disabilities. These understandings, however, are largely based on research assessing disability at one time point only, an approach that potentially obscures variation in disability over time. We aimed to identify trajectories of disability during childhood/adolescence and assess associations between trajectory membership and labor force status in young adulthood.

**Methods:**

We conducted group-based trajectory modeling of disability status information from six waves [waves 2–7 (age 4/5 to 16/17 years)] of the Longitudinal Study of Australian Children. The trajectories were used to predict labor force participation (employed, unemployed, not in the labor force) at wave 8 (18/19 years), adjusted for confounders.

**Results:**

We identified four trajectory groups of the prevalence of disability: low (75.5% of cohort), low increasing (9.7%), high decreasing (10.9%), and consistently high (3.9%). Individuals in the low increasing trajectory were nearly three times as likely to be unemployed at age 18/19 years compared to individuals in the low trajectory [risk ratio (RR) 2.96, 95% confidence interval (CI) 1.94–4.53]. Individuals in the consistently high trajectory had a greater RR of not being in the labor force at age 18/19 years compared to individuals in the low group (reference) (RR 3.65, 95% CI 2.21–6.02).

**Conclusions:**

Results suggest that prolonged and increasing experiences of disability among young Australians may be differentially associated with future labor force outcomes. Additional support to prepare young people for the labor force should focus on individuals who consistently or increasingly report a disability.

Disability is a common experience among young Australians, with nearly one in ten (9.3%) 15–24 year-olds estimated to have a disability, including sensory, cognitive, physical and psychosocial disabilities (1). Australian data show that unemployment rates among young people with disabilities (25%) are higher than both their peers without disabilities (11.5%) and the general working age population (4.6%) (2). Rates of participation in the labor force (ie, being employed or unemployed) are also lower among young Australians with disabilities: nearly half (48.5%) are not in the labor force compared to 28.4% of young people without disabilities (2).

These statistics are concerning as experiences of unemployment are associated with ongoing and future unemployment (3), a reduction in the quality of future work (4), and poorer physical and mental health (5, 6). Similarly, people with disabilities who are not in the labor force may have poorer mental health than their peers who are engaged in work (7). Time spent out of the labor force may exacerbate the barriers to entering the workforce, and therefore increase total time out of the labor force among young people with disabilities (8). As such, entering the labor force and gaining and maintaining employment when young is critical to establishing improved health and labor force outcomes in adult life.

Despite being a common experience, disability is a complex phenomenon. As defined by the International Classification of Functioning, Disability and Health (ICF), disability is the result of an interaction between a person’s health condition, environmental factors, and personal characteristics (9). Although previous research shows that young people with disabilities are more likely to be unemployed or not in the labor force than young people without disabilities, these studies typically assess disability at one time point [eg, (10, 11)]. Given that disability status may vary over time, treating disability as a fixed category may mask variation in the experience of disability among young people, and the individual and household factors that are characteristic of different patterns of disability. Additionally, changing disability status likely has ramifications for the barriers to gaining and maintaining work that young people with disabilities may experience. Individuals who acquire a disability during childhood may have different needs to enable labor force participation compared to peers who consistently report disabilities. Therefore, identifying patterns (or trajectories) of disability throughout childhood and adolescence and their associations with early labor force status will identify individuals who may benefit from enhanced supports and interventions to improve their education to work transition, and future working lives.

This study uses group-based trajectory modeling (GBTM) to assess how distinct trajectories of disability experienced from age 6/7 years to 16/17 years are associated with labor force status at age 18/19 years among young people in the Longitudinal Study of Australian Children (LSAC). The aims of this study were to: (i) identify trajectories of disability throughout childhood and adolescence (age 6/7 to 16/17 years); (ii) describe the characteristics of individuals within each trajectory of disability; and (iii) explore the relationship between these trajectories and labor force status at age 18/19 years.

## Methods

### Data source

Data for this study were taken from the nationally representative LSAC, which began in 2004. Participating families have been interviewed every two years, with the most recent wave of data, wave 8, collected in 2018. The selection of the baseline LSAC sample has been described in more detail elsewhere (12), but briefly a two-stage clustered sample design was employed, first selecting postcodes and then children enrolled in the Medicare Australia database. Apart from some remote areas, the sample was selected to be representative of all Australian children in each of the two selected age cohorts, cohort B (age ≤1 year in 2004) and cohort K (age 4–5 years in 2004) (13). LSAC is a rich data source, combining information from parents, teachers, and other caregivers and study children themselves when appropriate. LSAC participants and their families are interviewed once per wave, with data collection for the entire cohort conducted over an average period of 11 months (eg, wave 1 was collected from March 2004 to January 2005) (13). The Australian Institute of Family Studies Ethics Committee approved LSAC research methodology and survey content (wave 8 application number 17-01).

This analysis uses data from cohort K waves 1–8, encompassing ages 4/5 years to 18/19 years. Information reported by parents and the study child in face-to-face, computer assisted interviews, or self-completed surveys was used. A total of 4983 study children comprised the wave 1, cohort K participants (13).

### Measures

*Disability*. Disability status was ascertained at each wave from wave 2–7 using information provided by the primary parent informant (typically the child’s mother). The respondent was asked “Does the study child have a medical condition or disability that has lasted for 6 months or more?” If the reply was yes, the respondent was able to select from 11 different conditions, see appendix S1 in the supplementary material (www.sjweh.fi/article/3994).

There was slight variation in the disability question and included conditions over time. In waves 2–4, mental illness was not included as one of the disabilities or long-term conditions respondents could select. However, mental illness was asked about as a restriction to everyday activities. For waves 2–4, we therefore included individuals who reported a mental illness as a restriction in the disability variable. In waves 5–7, mental illness was included as one of the disabilities or long-term health conditions participants could select.

For this study, we have used a binary indicator of disability status (yes, no) at each wave to represent the study child’s disability status.

*Labor force status*. The participant reported labor force status at wave 8 (18/19 years of age). We coded their employment status into three categories using Australian Bureau of Statistics definitions (14). Individuals who reported that they worked ≥1 hours in a job, business, or farm in the last week were classified as employed. Young people who were not working in the last week but had been actively looking for work in the past four weeks and were available to start work were categorized as unemployed. Participants who were neither employed nor unemployed were not considered to be in the labor force.

### Confounders

The adjusted multinomial logistic regression model of the association between disability trajectory and labor force status included the following confounders: child gender (male, female), child indigenous status (not Aboriginal or Torres Strait Islander, Aboriginal and/or Torres Strait Islander), child speaks language other than English at home (no, yes), dual or single parent household (dual, single), highest parent year 12 completion (completed year 12, did not complete year 12), housing tenure [owned outright, mortgaged, rented, other (eg, occupied under life tenure scheme, living rent free, or other arrangement)], main household income source (wages, rental property, or dividends; government allowances, pension, or other income source), and mother’s age (years).

### Analysis

The GBTM analysis was reported following the Guidelines for Reporting on Latent Trajectory Studies checklist, see supplementary table S1 (15). Trajectories of disability were identified based on six waves of child disability data (waves 2–7). Individuals were included in the GBTM if they provided data on disability status in ≥1 wave from wave 2–7. Trajectories were identified using a logistic model (16) using survey weights to account for the survey design and non-response and to permit interpretation of the resulting trajectories as population prevalence (17). We allowed for possible non-linear shapes of the trajectories using polynomial transformations of time (based on wave) but limited the transformation to a quadratic shape to prevent overfitting the data. Nonsignificant (P>0.05) polynomial terms were excluded from the model (18). We considered models with between two and four trajectory groups. Optimum model selection was guided by the Bayesian Information Criterion (BIC), the mean posterior probabilities, and expert opinion of the authors (18, 19). Following the selection of the optimum trajectory model, individuals were assigned to a trajectory group based on their maximum posterior probability of group membership (18). We calculated the mean posterior probability of group membership and odds of correct classification (OCC) for each trajectory group to further assess model fit (18).

Following the identification of the trajectory groups, we used these groups as predictors of labor force status at wave 8. We used a multinomial logistic regression model to assess the association between trajectory group membership and labor force status at age 18/19 years, with results given as relative risk ratios (RR). The adjusted analysis included the confounders detailed above. Survey weights from wave 1 were included in the regression models (20).

To evaluate the potential impact of missing disability data on our results, we used univariate logistic regression to describe the association between the confounders and wave 2 disability status on missing disability data at follow-up (waves 3–7). Associations between confounders and missing disability data are shown in supplementary table S2. Results suggest that the following factors were associated with increased odds of missing data at follow-up: being Aboriginal or Torres Strait Islander, speaking a language other than English at home, living in a single parent household, having parents who did not complete year 12, living in rented or other kinds of housing, and living in a household where the main income source was from government allowances, pension, or other.

All analysis was performed using Stata version 16 (StataCorp, College Station, TX, USA). The traj plugin was used to perform the GBTM (21).

## Results

### Descriptive characteristics

Table 1 describes participant wave 1 characteristics (N=4983) and wave 8 labor force status (N=2602). The sample was evenly split amongst males and females, 3.7% of children were Aboriginal or Torres Strait Islander, 89% of children did not speak a language other than English at home, 86% lived in a household with two parents, and 68.3% had one or both parents who completed year 12. Over half of households (58.4%) had a mortgage and 27.8% were renting. Most (84.6%) of the sample’s main household income source was from wages, rental property, or dividends. Mother’s mean age was 34.6 years. At wave 8, 72.4% of participants were employed, 11.6% were unemployed, and 16% were not in the labor force. Supplementary table S3 shows the prevalence of disability by wave in the sample.

**Table 1:** Participant wave 1 characteristics (N=4983) and wave 8 labor force status (N=2602). [SD=standard deviation.]

	N	%	Mean	SD
Child gender				
Male	2536	50.9		
Female	2447	49.1		
Child indigenous status				
Not Aboriginal or Torres Strait Islander	4794	96.3		
Aboriginal or Torres Strait Islander	187	3.7		
Child speaks language other than English at home				
No	4359	89.0		
Yes	540	11.0		
Single/dual parent household				
Dual	4286	86.0		
Single	697	14.0		
Highest parent year 12 completion				
Completed year 12	3398	68.3		
Did not complete year 12	1581	31.7		
Housing tenure				
Owned outright	548	11.0		
Mortgaged	2907	58.4		
Rented	1384	27.8		
Other	135	2.7		
Household income: main income source				
Wages, from rental property, or dividends	4178	84.6		
Government allowance/pension or other ^a^	761	15.4		
Mother’s age (years)			34.6	5.3
Labor force status (wave 8)				
Employed	1883	72.4		
Unemployed	303	11.6		
Not in the labor force	416	16.0		

a Superannuation, child support, worker’s compensation

### Disability trajectory modeling

**Figure 1:**
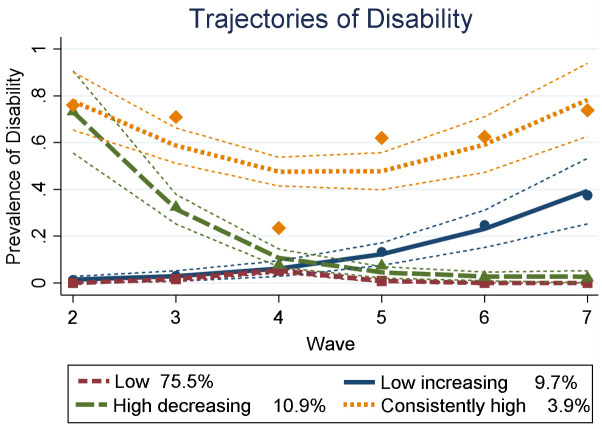
Estimated mean disability prevalence trajectories (N=4464) with 95% confidence intervals (thin dashed lines). The plotted curves show the estimated mean prevalence of disability as a function of study wave. The estimated population proportion in each trajectory group is displayed in the legend.

The best-fitting model identified four distinct trajectory groups (see supplementary table S4 for fit statistics). This model incorporated disability information from N=4464 participants with disability information reported at least once from waves 2–7. Supplementary table S5 presents coefficients for the estimated trajectories for each group. Figure 1 shows the groups as a function of time from childhood to adolescence. The groups included: low (75.5% of LSAC cohort), low increasing (9.7%), high decreasing (10.9%), and consistently high (3.9%) disability prevalence. For each group, the mean posterior probability and odds of correct classification were: low prob=0.90, odds=1.66; low increasing prob=0.88, odds=168.73; high decreasing prob=0.88, odds=77.2; consistently high prob=0.85, odds=188.77.

Table 2 shows participant characteristics according to trajectory group assignment. A total of N=3760 individuals were assigned to the low trajectory, N=191 to low increasing, N=381 to high decreasing, and N=132 to the consistently high trajectory. Individuals in the low trajectory were more likely than those in the high decreasing and consistently high groups to be female, and they were more likely – than the low increasing, high decreasing, and consistently high groups – to speak a language other than English at home, live in dual parent households, have ≥1 parent who completed year 12, and live in a household whose main income source was from wages, rental property, or dividends.

**Table 2:** Participant wave 1 characteristics according to trajectory group (N=4464).

	Low N=3760 (84.2%) ^a^	Mean	Low increasing N=191 (4.3%) ^a^	Mean	High decreasing N=381 (8.5%) ^a^	Mean	Consistently high N=132 (3.0%) ^a^	Mean	P-value
Child gender									<0.0001 ^b^
Male	49.5		40.3		62.7		75.8		
Female	50.5		59.7		37.3		24.2		
Child indigenous status									0.0707 ^b^
Not Aboriginal or Torres Strait Islander	96.6		97.4		94.8		99.2		
Aboriginal or Torres Strait Islander	3.4		2.6		5.3		0.08		
Child speaks language other than English at home									0.0002 ^b^
No	89.0		93.7		93.4		97.7		
Yes	11.0		6.3		6.6		2.3		
Single/dual parent household									0.0288 ^b^
Dual	87.9		85.3		85.3		80.3		
Single	12.2		14.7		14.7		19.7		
Highest parent year 12 completion									0.0043 ^b^
Completed year 12	70.7		68.1		62.7		62.9		
Did not complete year 12	29.3		31.9		37.3		37.1		
Housing tenure									0.6589 ^b^
Owned outright	11.5		9.4		11.3		6.8		
Mortgaged	60.7		60.7		57.2		62.9		
Rented	25.2		26.7		27.8		27.3		
Other	2.6		3.1		3.7		3.0		
Household income: main income source									0.0007 ^b^
Wages, from rental property, or dividends	87.0		86.4		83.4		75.8		
Government allowances or pension	13.0		13.6		16.6		24.2		
Mother’s age (years)		34.9		35.0		34.3		34.4	0.2032 ^c^

a Percentages relate to the proportion after weighting.

b P-values from Pearson Chi squared test incorporating survey weights.

c Estimated using univariate linear regression.

Participants in the high decreasing and consistently high trajectories were more likely to be male than individuals in the low and low increasing trajectories. Individuals in the consistently high trajectory were least likely to speak a language other than English at home, and they were most likely to live in a single parent household. A similar proportion of individuals in the high decreasing and consistently high groups had parents who did not complete year 12. Participants in the consistently high trajectory were most likely to live in a household whose main income source was from government allowances or pensions.

### Disability trajectories and labor force outcomes

Descriptive information regarding labor force status and disability trajectory can be seen in supplementary table S6. Results of the adjusted multinomial regression analysis assessing the association between disability trajectory and labor force status are shown in table 3. Individuals in the low increasing group were nearly three times as likely to be unemployed at age 18/19 years compared to individuals in the low trajectory (RR 2.96, 95% CI 1.94–4.53). The RR for unemployment at age 18/19 years for the high decreasing (1.09, 95% CI 0.68–1.74) and consistently high (1.36, 95% CI 0.66–2.82) trajectories are less conclusive as suggested by the wide CI that include the null value.

**Table 3:** Results of adjusted survey weighted multinomial logistic regression model: relative risk ratio (RR) for labor force status according to trajectory group membership (N=2535). [CI=confidence interval.]

	Labor force status				

	Employed	Unemployed		Not in the labor force	
		
	RR (95% CI)	RR (95% CI)	P-value	RR (95% CI)	P-value
Trajectory group					
Low	Reference	Reference		Reference	
Low increasing	Reference	2.96 (1.94–4.53)	<0.001	1.46 (0.87–2.45)	0.153
High decreasing	Reference	1.09 (0.68–1.74)	0.723	1.38 (0.91–2.09)	0.126
Consistently high	Reference	1.36 (0.66–2.82)	0.404	3.65 (2.21–6.02)	<0.001
Child gender					
Male	Reference	Reference		Reference	
Female	Reference	0.85 (0.66–1.09)	0.204	0.83 (0.66–1.05)	0.119
Child indigenous status					
Not Aboriginal or Torres Strait Islander	Reference	Reference		Reference	
Aboriginal or Torres Strait Islander	Reference	1.65 (0.76–3.59)	0.207	2.18 (1.03–4.62)	0.041
Child speaks language other than English at home					
No	Reference	Reference		Reference	
Yes	Reference	1.39 (0.87–2.20)	0.166	2.60 (1.87–3.63)	<0.001
Single/dual parent household					
Dual parent	Reference	Reference		Reference	
Single parent	Reference	0.80 (0.47–1.37)	0.416	0.88 (0.55–1.43)	0.615
Highest parent year 12 completion					
Complete year 12	Reference	Reference		Reference	
Did not complete year 12	Reference	0.97 (0.72–1.31)	0.843	0.70 (0.52–0.93)	0.013
Housing tenure					
Owned outright	Reference	Reference		Reference	
Mortgaged	Reference	0.71 (0.49–1.02)	0.061	0.78 (0.56–1.08)	0.134
Rented	Reference	1.03 (0.66–1.61)	0.895	1.27 (0.85–1.89)	0.246
Other	Reference	0.51 (0.21–1.26)	0.143	0.82 (0.40–1.69)	0.591
Household income: main income source					
Wages, from rental property, or dividends	Reference	Reference		Reference	
Government allowances or pension	Reference	1.78 (1.07–2.96)	0.026	1.08 (0.68–1.71)	0.754
Mother’s age (years)	Reference	1.00 (0.98–1.03)	0.880	1.04 (1.01–1.06)	0.002

Being in the consistently high trajectory was associated with a greater RR of not being in the labor force at age 18/19 years compared to individuals in the low trajectory (RR 3.65, 95% CI 2.21–6.02). The RR for individuals in the low increasing (1.46, 95% CI 0.87–2.45) and high decreasing (1.38, 95% CI 0.91–2.09) trajectories may suggest that young people in these groups have a greater probability of being not in the labor force compared to individuals in the low trajectory, although the CI are wide and include the null value.

## Discussion

Although the point prevalence of disability across young Australians is estimated to be 7.6% of children aged 0–14 years and 9.3% of young adults aged 15–24 years (2), the disability trajectories presented in this study suggest that about one-quarter of young Australians experience disability across the ages 6/7 to 16/17 years. The estimated population percentages from this study indicate that over 10% of young people experience either a consistently high or increasing prevalence of disability as they age.

The labor force outcomes of young Australians are associated with these disability trajectories. Individuals in the consistently high disability trajectory had increased risk of being not in the labor force at age 18/19 years. Young people in the low increasing trajectory, who may be acquiring new disabilities or experiencing an increase in limitations due to existing health conditions, had increased risk of being unemployed. One explanation for these results may be disability severity, as young people with consistent disabilities may be more likely to have severe disabilities that limit their labor force participation, potentially resulting in being permanently unable to work and receiving government benefits such as disability pensions. We note, however, that only 7% of those in the consistently high group were in receipt of a disability pension at age 18/19 years, suggesting that this is unlikely to account for the associations observed.

Parental expectations and work-related opportunities may also be decreased among young people with consistent disabilities, while young people in the low increasing group may have matured in the context of greater expectations of, and opportunities for, employment and work preparedness. Both expectations and opportunities are predictors of employment after secondary school among young people with disabilities (22), and may explain why young people in the consistently high trajectory have greater risk of being not in the labor force, while individuals in the low increasing group have greater risk of being unemployed.

For young people in the high decreasing group, the impact of disability trajectory on labor force status was less clear. This may arise from heterogeneity with regards to the types of conditions and the limitations young people in this group may experience, as a high decreasing disability prevalence may indicate a young person’s responses to medication or treatment, or the benefit of assistive devices and other disability supports in enabling greater labor force participation.

Males were more likely to be in the high decreasing and consistently high disability trajectories, while a greater proportion of females were in the low increasing trajectory. This aligns with evidence showing that disability is more common among males (9.5%) at ages 0–14 years than females (5.7%) (23), with prevalence increasing to 9.5% among females at ages 15–24 years, approximately equal to males (9.2%) (2). In general, individuals in the low trajectory came from more socioeconomically advantaged families, as captured by parent education, household income source and dual-parent family status. This likewise concurs with previous research which has shown that lower childhood socioeconomic status is predictive of onset and severity of mental illness among adults (24) and increases risk of future work disability (25). These results reinforce that disability is associated with socioeconomic status, although further research exploring the intricate relationship between time-varying socioeconomic indicators, disability trajectory, and future labor force outcomes could clarify groups of young people who are most in need of support and intervention throughout childhood and adolescence.

### Strengths and limitations

In interpreting the results of this study, there are several important limitations to be aware of. As with most panel studies of its kind, LSAC suffers from attrition. Young people who identified as Aboriginal or Torres Strait Islander, whose parents had lower levels of education, whose parents spoke a language other than English at home, and who lived in single-parent families were more likely to drop out of the study (20). This is consistent with patterns of non-response in the literature (26).

Additionally, there was slight variation in the disability questions included in LSAC over time, such as the inclusion of mental illness as either a restriction to everyday activities or as a disability or long-term health condition. While this study considered disability status as a dynamic phenomenon, we were unable to explore specific types of disability or disability severity due to small numbers of individuals in some groups, and the complex time-varying nature of disability type and severity. This approach may obscure heterogeneity with regards to disability type and severity within the disability trajectories, making it more difficult to identify individuals with the greatest needs for support. Furthermore, there were small numbers of individuals in some of the trajectories, namely the *consistently high* and *low increasing* groups, leading to reduced statistical power and wider confidence intervals around estimates. These small numbers also meant we were unable to examine labor force status at a more granular level, such as separating young people who were not in the labor force but studying from their peers who were not in employment, education, or training and who may particularly benefit from tailored interventions.

Finally, GBTM is based on defining ‘points of support’ for a continuous distribution of trajectories (18). Reality is more complex than the trajectories discussed in this paper suggest, and GBTM likewise does not reflect variation within disability groups. Similarly, the confounders we have included in our logistic regression models are a simplification of the complex interplay between disability status over time, socioeconomic status, and future labor force outcomes.

Strengths of this study include the use of an ongoing longitudinal dataset with rich socioeconomic, health, and labor force information that is representative of Australian young people. Additionally, this analytic approach among young people has not previously been done, and sheds light on the patterns of disability experienced by young people.

### Future research

Information on disability type and severity was not used in the construction of disability trajectories in the current study. Assessing variation within disability trajectory groups by type and severity could lead to more specific insights into the experience of disability throughout early life and its associations with labor force status during young adulthood. Methods such as growth mixture modeling could accommodate this information, but require substantial amounts of statistical power.

Additionally, the current study assessed the association between disability trajectory and labor force outcomes, but did not examine the quality of the employment that young people were entering. This is a key area for future analysis as people with disabilities may be more likely to experience poorer quality employment compared to their peers without disabilities (27). As further waves of LSAC become available, researchers will be able to assess how labor force outcomes evolve over time among young people with disabilities. Evaluating how the COVID-19 pandemic impacted the movement into the labor force of young people with and without disabilities will also be a key area of research. Future research could also explore the relationship between disability trajectory, labor force status, and mental health and wellbeing.

### Policy and practice implications

The barriers to finding and sustaining employment faced by the general population of young people [eg, lack of jobs, lack of work experience (28)] are being exacerbated by the COVID-19 pandemic (29). This may particularly be the case for young people with disabilities who face additional barriers to work including discrimination (30), lack of employer knowledge about how to accommodate disabilities in the workplace (31), and lack of adequate transition planning, including relevant career guidance and support (32). These barriers are not insurmountable: there is good evidence that work experience and vocational skills development while in secondary school, accompanied by appropriate transitions planning, predicts better post-school employment outcomes for young people with disabilities (33).

However, such programs have been found to be under-resourced and of poor and inconsistent quality, as identified by the Parliament of Victoria’s inquiry into career advice activities in schools (34). Young people experiencing disadvantage, including young people with disabilities, are particularly at risk of poor employment transitions due to these policy and programmatic failures. To this end, the Australian Government has launched a National Career Education Strategy to improve the quality of career education provided to young people (35). This strategy, if successful, has the potential to benefit all Australian young people, especially young people with disabilities.

Young people with disabilities who struggle to gain employment may be further disadvantaged by existing employment programs in Australia, which may be unsuitable for jobseekers with limited work experience and with disabilities (36). Given that employment is a human right for all (37), including people with disabilities as explicated in the United Nations Convention on the Rights of Persons with Disabilities (38), it is imperative to enact policies which actively facilitate improved labor force outcomes for young people as a whole, with a particular focus on young people who may experience greater disadvantage, such as young people with disabilities.

The results of this study have made clear that disability is a common experience, with over 10% of young people experiencing either a consistently high or increasing prevalence of disability as they age. The results have also demonstrated that the pattern of disability a young person experiences is related to their labor force outcomes, with individuals with an increasing or consistently high prevalence of disability having greater risk of being unemployed or out of the labor force altogether. This highlights the importance of understanding disability as a dynamic phenomenon among young people which has real impacts on their transition into the labor force. It underscores the urgent need for increased supports to help young people with and without disabilities successfully enter the labor force and gain and maintain employment.

## Supplementary material

Supplementary material

## Data Availability

LSAC unit record files are held by the Australian Data Archive at the Australian National University. Access to data is free via a formal request and registration with the ADA. Individuals can register and request data at this website: https://dataverse.ada.edu.au/dataverse/ncld.
