# Supplementary material for: Trajectories of disability throughout early life and labor force status as a young adult: Results from the Longitudinal Study of Australian Children
Source: Scand J Work Environ Health. 2022 Feb 25;48(2):118–26. doi: 10.5271/sjweh.3994 (PMC9045225; doi:10.5271/sjweh.3994)
Supplement: Supplementary material [file SJWEH-48-118-S001.pdf]

Trajectories of disability throughout early life and labor force status as a young adult: Results from the Longitudinal Study of Australian Children[1]

by Marissa Shields, MPH,[2] Matthew J Spittal, PhD, Stefanie Dimov, BPsychSc(Hons), Anne Kavanagh, PhD, Tania L King, PhD

1. *Supplementary material*
2. *Correspondence to: Marissa Shields, Centre for Health Equity, Melbourne School of Population and Global Health, The University of Melbourne, Victoria 3010 Australia. [E-mail: marissa.shields@unimelb.edu.au]*

Supplementary Table S1: Guidelines for Reporting on Latent Trajectory Studies (GRoLTS) checklist

1. Is the metric of time used in the statistical model reported?	Yes; methods
2. Is information presented about the mean of variance of time within a wave?	Yes; methods. We have reported the duration of data collection for the survey.
3a. Is the missing data mechanism reported?	Yes, Supplementary Table S7
3b. Is a description provided of what variables are related to attrition/missing data?	Yes; Supplementary Table S2
3c. Is a description provided of how missing data in the analyses were dealt with?	Yes; methods
4. Is information about the distribution of the observed variables included?	Yes, Supplementary Table S3
5. Is the software mentioned?	Yes; methods
6a. Are alternative specifications of within-class heterogeneity considered (e.g., LGCA vs. LGMM) and clearly documented? If not, was sufficient justification provided as to eliminate certain specifications from consideration?	Yes; we had considered using GMM but decided GBTM was appropriate for this exploratory analysis. See Appendix 2.
6b. Are alternative specifications of the between-class differences in variance-covariance matrix structure considered and clearly documented? If not, was sufficient justification provided as to eliminate certain specifications from consideration?	No; not an option in the available software.
7. Are alternative shape/functional forms of the trajectories described?	Yes, Supplementary Table S4
8. If covariates have been used, can analyses still be replicated?	NR
9. Is information reported about the number of random start values and final iterations included?	NR

10. Are the model comparison (and selection) tools described from a statistical perspective?	Yes; methods
11. Are the total number of fitted models reported, including a one-class solution?	Yes; Supplementary Table S4
12. Are the number of cases per class reported for each model (absolute sample size, or proportion)?	Yes; Supplementary Table S4
13. If classification of cases in a trajectory is the goal, is entropy reported?	Yes; Supplementary Table S4
14a. Is a plot included with the estimated mean trajectories of the final model?	Yes; Figure 1
14b. Are plots included with the estimated mean trajectories for each model?	Yes, Supplementary Figures S1a-S1d
14c. Is a plot included of the combination of estimated means of the final model and the observed individual trajectories split out for each latent class?	No, unsuitable for a binary variable.
15. Are characteristics of the final class solution numerically described (i.e., means, SD/SE, *n*, CI, etc)?	Yes; Results
16. Are the syntax files available (either in the appendix, supplementary materials, or from the authors)?	Yes; Available from authors upon reasonable request

Supplementary Table S2: Associations between wave 1 characteristics, wave 2 disability and missing disability data at one or more waves at follow-up*

Baseline characteristic	Odds ratio (95% CI)
Disability	
No disability	ref
Disability	1.14 (0.94, 1.38)
Child gender	
Male	ref
Female	0.95 (0.84, 1.07)
Child Indigenous Status	
Not Aboriginal or Torres Strait Islander	ref
Aboriginal or Torres Strait Islander	3.01 (2.15, 4.22)
Child speaks language other than English at home	
No	ref
Yes	1.92 (1.58, 2.34)
Single/dual parent household	
Dual parent	ref
Single parent	2.40 (2.01, 2.87)
Highest parent year 12 completion	
Completed year 12	ref

Did not complete year 12	2.02 (1.78, 2.30)
Housing tenure	
Owned outright	ref
Mortgaged	1.18 (0.96, 1.45)
Rented	2.32 (1.86, 2.90)
Other	1.70 (1.14, 2.55)
Household income: main income source	
Wages, from rental property, or dividends	ref
Government allowances/pension or other income source (superannuation, child support, worker's compensation)	2.73 (2.29, 3.25)
Mother's age (years)	0.94 (0.93, 0.95)

*From wave 3 to wave 7

Supplementary Table S3: Survey weighted prevalence of disability at waves 2-7

	Wave 2<br>N=4 464	Wave 3<br>N=4 331	Wave 4<br>N=4 169	Wave 5<br>N=3 913	Wave 6<br>N=3 454	Wave 7<br>N=3 041
No disability	89.2%<br>(88.3, 90.1)<br>N=3 982	92.5%<br>(91.7, 93.3)<br>N=4 011	94.0%<br>(93.3, 94.7)<br>N=3 914	95.3%<br>(94.5, 95.9)<br>N=3 733	95.0%<br>(94.2, 95.7)<br>N=3 283	93.5%<br>(92.6, 94.3)<br>N=2 848
Disability	10.8%<br>(9.9, 11.7)<br>N=482	7.5%<br>(6.7, 8.3)<br>N=320	6.0%<br>(5.3, 6.8)<br>N=255	4.7%<br>(4.1, 5.5)<br>N=180	5.0%<br>(4.3, 5.8)<br>N=171	6.5%<br>(5.7, 7.5)<br>N=193

Supplementary Table S4: Fit statistics for selecting model

	Number of groups			
	1	2	3	4
BIC	-5682.79	-5159.54	-5118.84	-5077.57
Mean Posterior Probability				
Group 1	1.0	0.97	0.97	0.88
Group 2		0.86	0.71	0.90
Group 3			0.84	0.88
Group 4				0.85
Sample size per class based on most likely class membership	4 464	4 103/361	4 109/195/160	191/3760/381/132
Entropy	1.0	0.964	0.955	0.895

The Bayesian Information Criterion (BIC) is calculated as: BIC=log(L)-0.6k log(N), where L is the model's maximized likelihood, N is the sample size, and k is the number of parameters in the model (1).

The highest order polynomial was dropped if it was not significant ($p<0.05$). We repeated this process until all higher order terms were significant.

Entropy reflects the quality of the classification of the model, with values closer to 1.0 indicating better classification. Entropy is calculated by averaging the posterior probabilities after individuals have been assigned to their most likely trajectory. Values may range from 0 to 1 (2).

Supplementary Table S5: Coefficients from the selected trajectory model with four groups

Polynomial term	Trajectory group											
	Low			Low increasing			High decreasing			Consistently high		
	Coefficient	SE	p value	Coefficient	SE	p-value	Coefficient	SE	p-value	Coefficient	SE	p-value
Intercept	-3.43	0.16	<0.001	-2.34	0.25	<0.001	-2.64	0.26	<0.001	-0.15	0.14	0.2969
Linear	-4.57	0.99	<0.001	1.91	0.33	<0.001	-2.30	0.28	<0.001	0.02	0.24	0.9503
Quadratic	-9.40	1.78	<0.001	-	-	-	1.34	0.49	0.0062	1.42	0.37	0.0002

Supplementary Table S6: Wave 8 labor force status by disability trajectory group

	Low N=2 171	Low increasing N=138	High decreasing N=207	Consistently high N=86	Total
Employed	74.2% (72.2, 76.0) N=1 610	59.4% (51.0, 67.3) N=82	70.5% (63.8, 76.5) N=146	52.3% (42.0, 62.4) N=45	72.4% (70.6, 74.1) N=1 883
Unemployed	10.8% (9.6, 12.2) N=235	25.4% (18.7, 33.4) N=35	11.1% (7.5, 16.2) N=23	11.6% (6.4, 20.2) N=10	11.7% (10.5, 12.9) N=303
Not in the labor force	15.0% (13.5, 16.7) N=326	15.2% (10.0, 22.5) N=21	18.4% (13.5, 24.5) N=38	36.1% (26.6, 46.7) N=31	16.0% (14.6, 17.5) N=416

*Percentages relate to the proportion after weighting

Supplementary Table S7: Patterns of non-response for 4 983 participants in the LSAC K Cohort

Variable	N	Percentage of original sample
Missing at each wave		
Wave 1	0	100.0
Wave 2	519	10.4
Wave 3	652	13.1
Wave 4	814	16.3
Wave 5	1 027	20.6
Wave 6	1 446	29.0
Wave 7	1 894	38.0
Wave 8	1 946	39.1
Missing for number of waves		
No missing waves	2 510	50.4
1 wave only	645	12.9
2 waves	473	9.5
3 waves	342	6.9
4 waves	248	5.0
5 waves	226	4.5
6 waves	214	4.3
7 waves	325	6.5
Patterns of non-response		
All complete	2 510	50.4
Wave 8 only	282	5.7
Wave 7 only	186	3.7
Wave 7 & 8	298	6.0
Wave 6 only	67	1.3
Wave 6 & 8	22	0.4
Wave 6 & 7	59	1.2
Wave 6, 7 & 8	258	5.2

Wave 5 only	20	0.4
Wave 5 & 8	7	0.1
Wave 5 & 7	9	0.2
Wave 5, 7 & 8	11	0.2
Wave 5 & 6	6	0.1
Wave 5, 6 & 8	6	0.1
Wave 5, 6 & 7	11	0.2
Wave 5, 6, 7 & 8	188	3.8
Wave 4 only	23	0.5
Wave 4 & 8	5	0.1
Wave 4 & 7	4	0.1
Wave 4, 7 & 8	8	0.2
Wave 4 & 6	5	0.1
Wave 4, 6 & 8	2	0.04
Wave 4, 6 & 7	3	0.06
Wave 4, 6, 7 & 8	20	0.4
Wave 4 & 5	5	0.1
Wave 4, 5 & 8	3	0.06
Wave 4, 5 & 7	3	0.06
Wave 4, 5, 7 & 8	3	0.06
Wave 4, 5, 6 & 7	1	0.02
Wave 4, 5, 6, 7 & 8	171	3.4
Wave 3 only	23	0.5
Wave 3 & 8	3	0.06
Wave 3 & 7	4	0.1
Wave 3, 7 & 8	7	0.1
Wave 3 & 6	3	0.06
Wave 3, 6 & 8	3	0.06
Wave 3, 6 & 7	1	0.02
Wave 3, 6, 7 & 8	9	0.2
Wave 3, 5 & 8	1	0.02
Wave 3, 5 & 7	1	0.02
Wave 3, 5, 7 & 8	1	0.02
Wave 3, 5, 6, 7 & 8	17	0.3
Wave 3 & 4	2	0.04
Wave 3, 4 & 8	1	0.02
Wave 3, 4, & 7	1	0.02
Wave 3, 4, 7 & 8	3	0.06
Wave 3, 4, 6, 7 & 8	1	0.02
Wave 3, 4, 5, & 6	1	0.02
Wave 3, 4, 5, 6 & 7	1	0.02
Wave 3, 4, 5, 6, 7 & 8	178	3.6
Wave 2 only	44	0.9
Wave 2 & 8	11	0.2
Wave 2 & 7	8	0.2
Wave 2, 7 & 8	10	0.2

Wave 2 & 6	1	0.02
Wave 2, 6 & 8	2	0.04
Wave 2, 6 & 7	4	0.1
Wave 2, 6, 7 & 8	17	0.3
Wave 2, 5 & 8	1	0.02
Wave 2, 5, 6 7 & 8	12	0.2
Wave 2 & 4	2	0.04
Wave 2, 4, 6, 7 & 8	2	0.04
Wave 2, 4 & 5	1	0.02
Wave 2, 4, 5 & 8	1	0.02
Wave 2, 4, 5, & 7	1	0.02
Wave 2, 4, 5, 6 & 7	1	0.02
Wave 2, 4, 5, 6, 7 & 8	17	0.3
Wave 2 & 3	19	0.4
Wave 2, 3 & 8	1	0.02
Wave 2, 3 & 7	2	0.04
Wave 2, 3, 7 & 8	1	0.02
Wave 2, 3, 6, 7 & 8	7	0.1
Wave 2, 3, 5, 7 & 8	3	0.06
Wave 2, 3, 5 6 & 8	1	0.02
Wave 2, 3,5, 6, 7 & 8	12	0.2
Wave 2, 3, 7 & 4	1	0.02
Wave 2, 3, 4, 7 & 8	1	0.02
Wave 2, 3, 4, 6, 7 & 8	4	0.08
Wave 2, 3, 4 & 5	2	0.04
Wave 2, 3, 4, 5 & 8	1	0.02
Wave 2, 3, 4, 5, 7 & 8	2	0.04
Wave 2, 3, 4, 5 & 6	1	0.02
Wave 2, 3, 4, 5, 6 & 7	1	0.02
Wave 2, 3, 4, 5, 6, 7 & 8	325	6.5

Figure S1a: 1 class solution

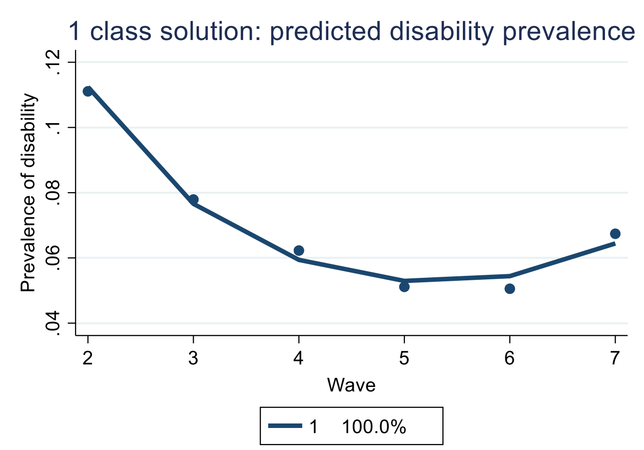

Figure S1b: 2 class solution

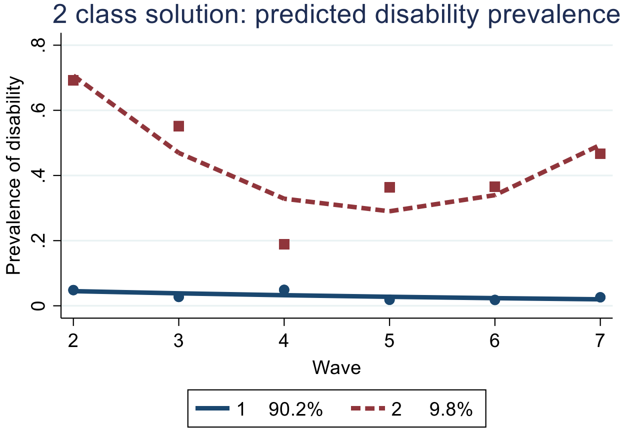

Figure S1c: 3 class solution

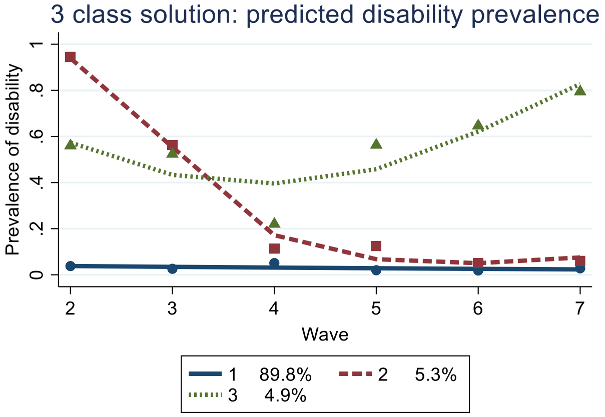

Figure S1d: 4 class solution

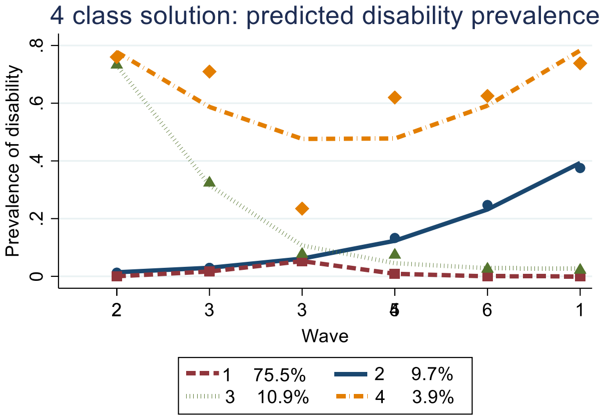

Appendix S1: Disability questions in LSAC

**Wave 2**	Does the study child have a medical condition or disability that has lasted for 6 months or more?	
If yes:		Which medical conditions or disabilities does the study child have?
		Sight problems not corrected by glasses or contact lenses<br>Hearing problems<br>Speech problems<br>Blackouts, fits, or loss of consciousness<br>Difficulty learning or understanding things<br>Limited use of arms or fingers<br>Difficulty gripping things<br>Limited use of legs or feet<br>Any condition that restricts physical activity or physical work (other physical condition)<br>Any disfigurement or deformity<br>None of the above conditions (i.e. some other condition)
To make wave 2 comparable with waves 5-7 we also included the item:		
	Which restrictions does the study child have?	
		Any mental illness for which help or supervision is required long-term

**Waves 3 & 4**	Does the study child have a medical condition or disability that has lasted, or is likely to last, for 6 months or more?	
If yes:		Which medical conditions or disabilities does the study child have?
		Loss of sight (not corrected by glasses or contact lenses)<br>Loss of hearing (where communication is restricted, or an aid to assist with, or substitute for hearing, is used)<br>Speech difficulties<br>Blackouts, fits, or loss of consciousness<br>Difficulty learning or understanding<br>Limited use of arms or fingers<br>Difficulty gripping or holding things<br>Limited use of legs or feet<br>Restriction in physical activities or in doing physical work<br>Disfigurement or deformity<br>None of the above conditions (i.e. some other condition)
To make waves 3 & 4 comparable with waves 5-7 we also included the item:		
	Which restrictions does the study child have?	
		Mental illness or condition requiring help or supervision

**Waves 5, 6 &7**	Does the study child have a medical condition or disability that has lasted for 6 months or more?	
If yes:		Which medical conditions or disabilities does the study child have?
		Sight problems not corrected by glasses or contact lenses<br>Hearing problems<br>Speech problems<br>Blackouts, fits, or loss of consciousness

	Difficulty learning or understanding<br>Limited use of arms or fingers<br>Difficulty gripping things<br>Limited use of legs or feet<br>Any condition that restricts physical activity or physical work (e.g. back problems, migraines)<br>Disfigurement or deformity<br>Any mental illness for which help or supervision is required

Appendix 2: Brief description of GBTM

GBTM assumes that the population is composed of distinct groups, each with a different underlying trajectory. These trajectory groupings represent individuals who follow similar developmental courses on the outcome of interest, (3) in this case disability. The groups can then be further explored to identify differences in baseline characteristics or subsequent outcomes (2).

To apply GBTM, the modeler must specify the number of trajectory groupings, and the trajectories themselves are then estimated parametrically directly from the data (4). Unlike methods such as growth mixture modeling (GMM) which incorporate random effects in each group's trajectory model, GBTM does not estimate within-group variation (2). However, GBTM approximates a more complex distribution of trajectories and is appropriate for exploratory analysis as it is less computationally demanding, involves fewer assumptions, and the results are more straightforward to interpret (2).

References

1. Nagin D. Group-Based Modeling of Development. Cambridge: Harvard University Press; 2005.
2. Nagin DS, Odgers CL. Group-based trajectory modeling in clinical research. Annu Rev Clin Psychol. 2010;6:109–38.
3. Nagin DS. Group-based trajectory modeling: An overview. Ann Nutr Metab. 2014;65:205–10.
4. Nagin DS. Analyzing developmental trajectories: A semiparametric, group-based approach. Psychol Methods. 1999;4(2):139–57.
